# Ultrastructure of precapillary sphincters and the neurovascular unit

**DOI:** 10.1530/VB-23-0011

**Published:** 2023-12-01

**Authors:** Søren Grubb

**Affiliations:** 1 Department of Neuroscience and Center for Translational Neuromedicine, Faculty of Health Sciences, University of Copenhagen, Copenhagen, Denmark

**Keywords:** precapillary sphincter, mural cells, endothelia, astrocyte, neurovascular coupling

## Abstract

Neurons communicate with vasculature to regulate blood flow in the brain, a process maintained by the neurovascular unit (NVU). This interaction, termed neurovascular coupling, is believed to involve astrocytes or molecules capable of traversing the astrocytic endfeet. The precise mechanism, however, remains elusive. Using large 3D electron microscopy datasets, we can now study the entire NVU in context of vascular hierarchy. This study presents evidence supporting the role of precapillary sphincters as a nexus for neurovascular coupling and endothelial transcytosis. It also highlights the role of fibroblast-synthesized collagen in fortifying first-order capillaries. Furthermore, I demonstrate how astrocytic endfeet establish a barrier for fluid flow and reveal that the cortex’s microvasculature is semicircled by an unexpected arrangement of parenchymal neuronal processes around penetrating arterioles and arterial-end capillaries in both mouse and human brains. These discoveries offer insights into the NVU’s structure and its operational mechanisms, potentially aiding researchers in devising new strategies for preserving cognitive function and promoting healthy aging.

## Introduction

The neurovascular unit (NVU) is a complex structure that orchestrates the blood-oxygen level dependent (BOLD) response, a mechanism linking neural activity to alterations in blood flow (1). Comprising endothelial cells, vascular mural cells, glial cells, and neurons, the NVU has been the subject of extensive research (1, 2, 3, 4, 5). However, our understanding of their structures and interactions remains incomplete.

Unraveling the communication dynamics among these cells and identifying the aberrations during the progression of brain diseases is paramount for devising effective treatments to halt disease advancement.

Neurovascular coupling (NVC), the mechanism behind the BOLD response, is a process that has intrigued scientists for over a century and is a key area of interest. As August Krogh pondered in his Nobel Prize lecture, ‘In what way can the capillaries be excited – chemical, electrical, or mechanical? Are they under nervous control, and, if so, by which nerve?’ (6).

In collaboration with my colleagues, I have recently delineated the structure, location, and function of brain precapillary sphincters (7), integral components of the NVU. These sphincters form a bottleneck for blood flow to the capillary bed and assist in distributing blood flow across the cortical layers. Johannes Rhodin’s pioneering work on the ultrastructure of precapillary sphincters in the rabbit thigh muscle fascia (8) over half a century ago inspired the present study. However, the ultrastructure of brain precapillary sphincters remains largely unexplored. The precapillary sphincter is an important part of the ‘resistance microvasculature’, the microvascular part of the resistance vasculature, including arterioles and low-order capillaries (precapillaries) that protect capillaries from harmful blood pressures, but also provides a resistance to flow, allowing for redistribution of blood flow to active brain areas facilitated by NVC (2). Therefore, to investigate the NVU of precapillary sphincters ultrastructurally, it must be studied in the context of vascular hierarchy.

In this study, I introduce novel findings on the precapillary sphincter and NVU’s ultrastructure. The focus of the present study is on the ultrastructure of the NVU of brain precapillary sphincters but to put it in context of vascular hierarchy I will compare it to arterioles, venules, and capillaries. This study of the 3D EM dataset uncovers the NVU of precapillary sphincters, a unique organization of neuronal processes around resistance microvasculature and the role of astrocytic endfeet in establishing a barrier to the parenchyma.

## Materials and methods

### Public volume electron microscopy datasets

The primary dataset used in this study is the MICrONS dataset, provided by the Allen Institute (9). This dataset comprises approximately 1 mm^3^ of the male mouse’s (P75–87) visual cortex, including parts of the primary visual cortex and higher visual areas for all cortical layers excluding the extremes of L1. The dimensions are (*in vivo*) 1.3 mm mediolateral, 0.87 mm anterior–posterior and 0.82 mm radial. The dataset’s resolution was initially ~4 nm but was downsampled to 8 nm/pixel, with a slice thickness of 40 nm. The dataset is visualized using the Neuroglancer web-based application developed by Google. A guide to navigating the tool can be found on the MICrONS website: microns-explorer.org/visualization. Unique codes that identify each segment and 3D locations (x, y, z) can be copied or pasted into Neuroglancer for reference. In addition to the MICrONS dataset, the public H01-release dataset was used to confirm the presence of certain findings in human data (10). This dataset includes a temporal lobe cortical fragment from a 45-year-old patient with drug-resistant epilepsy. However, it should be noted that the vascular lumen in this dataset is collapsed (11), and the astrocyte endfeet are swollen, making it difficult to characterize vascular cells.

### Quantification

The quantification of pinocytic vesicles was conducted using the point annotation tool in Neuroglancer. I selected twelve penetrating arteriole (PA) branch points based on their branchpoint-to-first-order capillary lumen ratio (refer to Supplementary Table 1, see section on supplementary materials given at the end of this article). Of these, six had a precapillary sphincter, while the other six did not have a sphincter at the branchpoint. It was ensured that the vessel’s cross section was parallel to the imaging plane. I gave priority to those with pronounced lumen indentations, specifically those with the smallest ratio of branchpoint diameter to first-order capillary diameter. The final quantification was derived from the average count across three images, each separated by 400 nm, situated immediately downstream of the PAs’ branchpoints. The count was normalized to cytosol area, which was measured in ImageJ.

The quantification of myoendothelial junctions was conducted similarly, but instead of averaging three images, I counted all myoendothelial junctions within a bounding box reaching across 20 consecutive images with a total of 800 nm thickness. The count was normalized to endothelial surface area, which was calculated by measuring the length of the endothelial plasma membrane in one plane and multiplying it by 800 nm.

### ImageJ and OrientationJ analysis

The orientation of neuronal processes was analyzed using the OrientationJ plugin for ImageJ. Screenshots from four different views of the parenchymal perivascular wall were taken, excluding astrocytes. The screenshots were cropped to the diameter of the parenchymal perivascular wall, changed to 8 bits using ImageJ, and analyzed using OrientationJ. The orientation of neuronal fibers in the perivascular wall was visualized, and the distribution was quantified. Positive and negative angles were averaged to find the angles different from the vessel direction.

## Results

This study examined a large publicly available 3D electron microscopy (EM) dataset of approximately 1 mm³ of the visual cortex of a male mouse at 2.9 months of age, 23 days after implantation of a cranial window over the visual cortex (9) (https://n9.cl/vid4i). The dataset contains 20 PAs and 21 precapillary sphincters were identified among 95 scrutinized PA branchpoints. The determination was made by contrasting the lumen diameter of the capillary at its arteriolar branchpoint with that of the first-order capillary. A branchpoint was identified as a precapillary sphincter if its diameter was less than 80% of the first-order capillary’s diameter, consistent with prior definitions (7). The majority of these were located within the first three capillary branches of the PAs (Supplementary Table 1).

To grasp the significance of precapillary sphincters within the NVU, it is essential to closely examine the ultrastructure of cell types that impact the function of these sphincters, taking into consideration the broader framework of the resistance microvasculature. In the following, I will detail the ultrastructure of the cells constituting the NVU, ranging from mural cells, endothelial cells and fibroblasts that form the vasculature to astrocytes and neurons that form the brain parenchyma, and compare their ultrastructure with current knowledge about these cell types.

### Vascular mural cells exhibit a continuum of shapes at the resistance microvasculature

**Figure 1:**
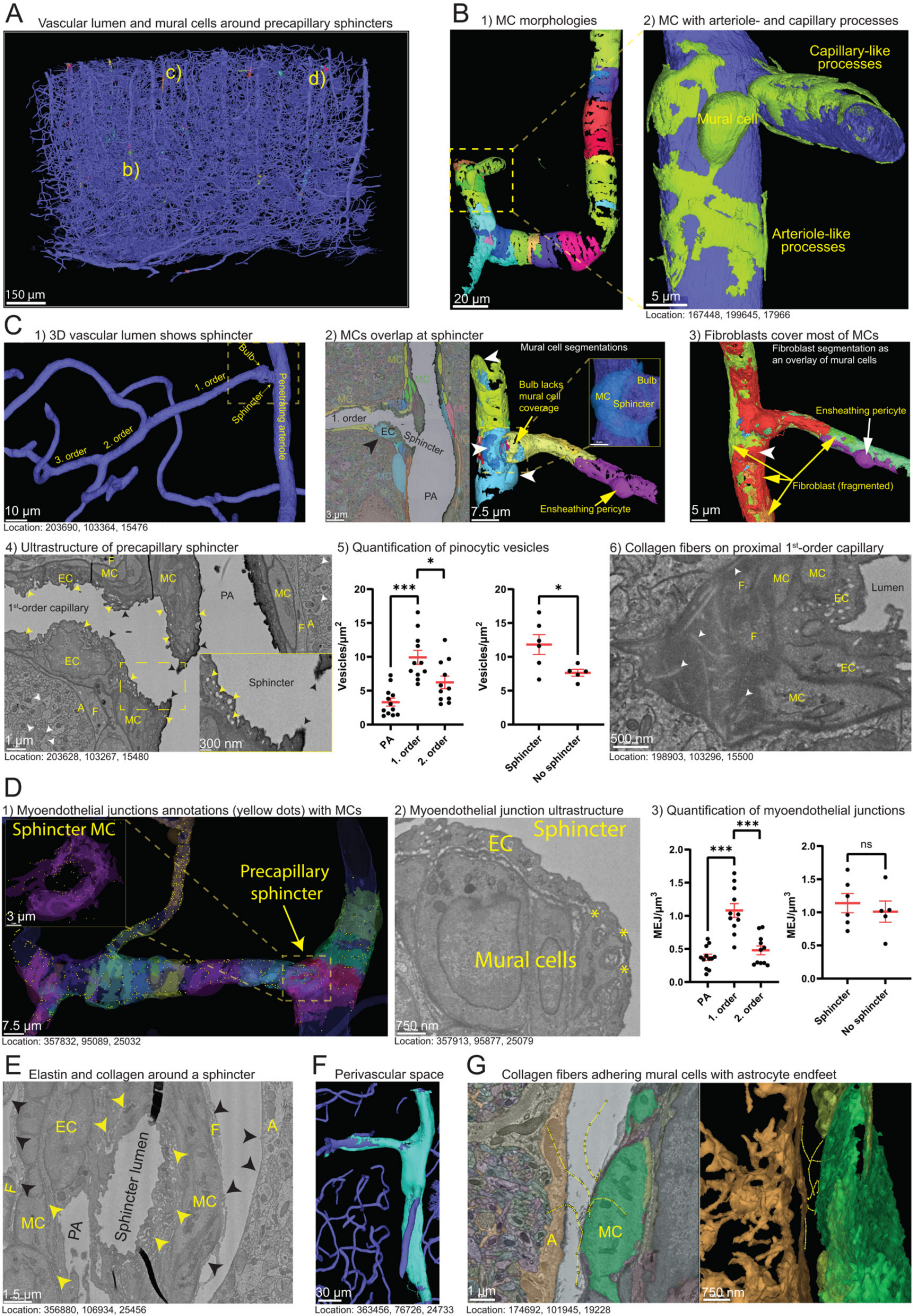
Ultrastructure of the precapillary sphincter. (A) Overview of the vasculature lumen in the dataset, highlighting visible mural cells around precapillary sphincters. Yellow letters (b, c, d) indicate the locations of vessels depicted in Figure 1B, C, and D. (B) 1) Mural cells around a PA are not simple bands but feature multiple processes encircling the arteriole. 2)Mural cells positioned on both the arteriole and capillary exhibit processes characteristic of arteriolar mural cells and ensheathing pericytes. (C) 1) 3D segmentation of vascular lumen of a PA with first- to third-order capillaries. Dashed lines indicate the area depicted in the panels to the right. 2) Combined EM and 3D segmentation of mural cells around the PA branchpoint. Black arrowhead indicates an endothelial nucleus lacking mural cell coverage. White arrowheads point to mural cell somas. Inset shows semitransparent segmentations of the vascular lumen and the mural cell encircling the precapillary sphincter. 3) 3D segmentation of a fibroblast with its soma (white arrowhead) near the branchpoint. The yellow, red, and green segmentation are all a part of the same fibroblast. The fibroblast partly covers the ensheathing pericyte on the first-order capillary. 4) Ultrastructure of the precapillary sphincter, illustrating how layers of mural cell (MC) processes encircle the indentation, contrasting with a single layer on the PA and on the first-order capillary. It also shows how the endothelial cell (EC) nucleus protrudes into the lumen and its basement membrane is in contact with a fibroblast (F) and astrocytic (A) endfeet on the abluminal side. The glycocalyx (black arrowheads) is thick and dark on the PA, but lighter and thinner downstream of the precapillary sphincter. The number of EC micropinocytic vesicles is higher immediately downstream of the precapillary sphincter than in the PA. Synapses (white arrowheads) also exist near the vasculature. Inset shows an enlargement of the precapillary sphincter area (dashed yellow lines). See also Supplementary Video 2. 5) Quantification of pinocytic vesicles in endothelial cells at PAs, first-order capillaries or second-order capillaries and for first-order capillaries with or without a precapillary sphincter. Each data point is an average of three counts spaced 400 nm apart and is normalized to endothelial cytosol area and shown as mean ± s.e.m. for six branchpoints with and six branchpoints without a precapillary sphincter. One outlier (~18 vesicles/µm^2^) was removed for the no-sphincter data. 6) Collagen fibrils (white arrowheads) can be seen in the extracellular matrix abluminal of mural cells at the proximal end of first-order capillaries. (D) 1) 3D segmentation of a PA with a precapillary sphincter and first- to third-order capillaries. The yellow dots represent individual myoendothelial junctions (or peg-and-socket). Inset shows an enlargement of the mural cell encircling the precapillary sphincter. See also Supplementary Video 3. 2) Ultrastructure of the EC and mural cells of the precapillary sphincter showing three myoendothelial junctions (*). 3) Quantification of myoendothelial junctions (MEJ) at the same locations as in C. Total number of MEJs were counted within a bounding box of 800 nm thickness and the width and height of the vasculature. One outlier was removed for the first-order capillary data (~2.8 MEJ/µm^2^) and one for the second-order capillary data (~1.75 MEJ/µm^2^). (E) Precapillary sphincters have a high amount of elastin (yellow arrowheads) between the endothelial cells (EC) and the mural cells (MC) and collagen fibrils (black arrowheads) between the mural cells and fibroblasts (F). Collagen fibrils are also present in the PVS on the luminal side of astrocyte (A) endfeet. (F) 3D segmentation of the PVS (turquoise) and the vascular lumen (dark blue). The PVS reaches from the surface of the brain deep into the cortex along PAs but stops at first-order capillaries. (G) Tracing of collagen fibrils (yellow lines with dots) crossing the PVS from the surface of mural cells (green) to the astrocyte endfeet (orange). Quantification data were tested by Student’s *t*-test and corrected for multiple comparisons by doubling the *P*-value (*: *P* < 0.05, ***: *P* < 0.001).

Mural cells form the second layer of the blood vessel wall, encapsulated by a basement membrane and abluminal to endothelial cells. The term ‘mural cell’ encompasses both vascular smooth muscle cells and pericytes that exist on arterioles, capillaries, and venules of microvasculature. The mural cells of the resistance microvasculature are the contractile part of the NVU. The 3D ultrastructure of mural cells of the resistance microvasculature reveals a transition of morphologies (2) from arterioles to capillaries dependent on vascular lumen diameter (Figs. 1B and 4A). At the level of thin-strand pericytes and venular smooth muscle cells, the MICrONS provided segmentation is often erroneous and results in the amalgamation of pericytes with endothelial cells (Supplementary Fig. 1C).

Precapillary sphincters, encircled by contractile mural cells (Fig. 1C2, Supplementary Video 1), are predominantly found at proximal branches of arterioles in the cortex’s upper layers (7), which is consistent with this dataset (Supplementary Table 1). However, the dataset also contains a precapillary sphincter at a white-matter arteriole (Supplementary Fig. 1D). Intracellular structures of mural cells can be examined in the MICrONS dataset. Yet no discernible differences seem to exist between the ultrastructure of mural cells surrounding the precapillary sphincter and those around arterioles, and both may have processes encircling the precapillary sphincter.

The contractile apparatus of arteriolar and precapillary sphincter mural cells (8) is visible as faint intracellular lines traversing the cells (Supplementary Fig. 1A1–2) but becomes increasingly difficult to identify as the capillaries order increases, in line with an absence of contractile activity. In contrast to the mural cells of arterioles and precapillary sphincters, the cytosol of ensheathing and thin-strand pericytes frequently appears more electron-dense (as evidenced in these rare examples of ensheathing pericytes situated abluminally to contractile mural cells, location: 232043, 182475, 19109 and Fig. 1B). Thin-strand pericytes in the MICrONS dataset have been described in the study by Bonney *et al.* (11) and will not be detailed further in this work.

Precapillary sphincter and arteriolar mural cells have abundant vesicles and/or caveolae at the luminal and abluminal plasma membrane (Supplementary Fig. 1A3 and B), while at capillaries, like previously reported (12), they exist primarily on the abluminal side (Supplementary Fig. 1B). The luminal vesicles may have a role in elastin secretion (Supplementary Fig. 1A3), a component of the elastic lamina of arteries and arterioles synthesized by mural cells. The elastic lamina provides an elasticity that enables the vasculature to dampen pulse-pressure waves traveling from the heart to the microcirculation, known as the ‘windkessel effect’. In the MICrONS dataset, elastin is identified as an electron-lucent core with electron-dense borders of fibrillin at arterioles and at precapillary sphincters (Fig. 1D2, E and Supplementary Fig. 2A2), but not beyond that point, consistent with Alexa Fluor 633 hydrazide staining (7, 13). Interestingly, a mural cell with a rudimentary primary cilium facing the elastic lamina was observed (Supplementary Fig. 1A3).

**Figure 2:**
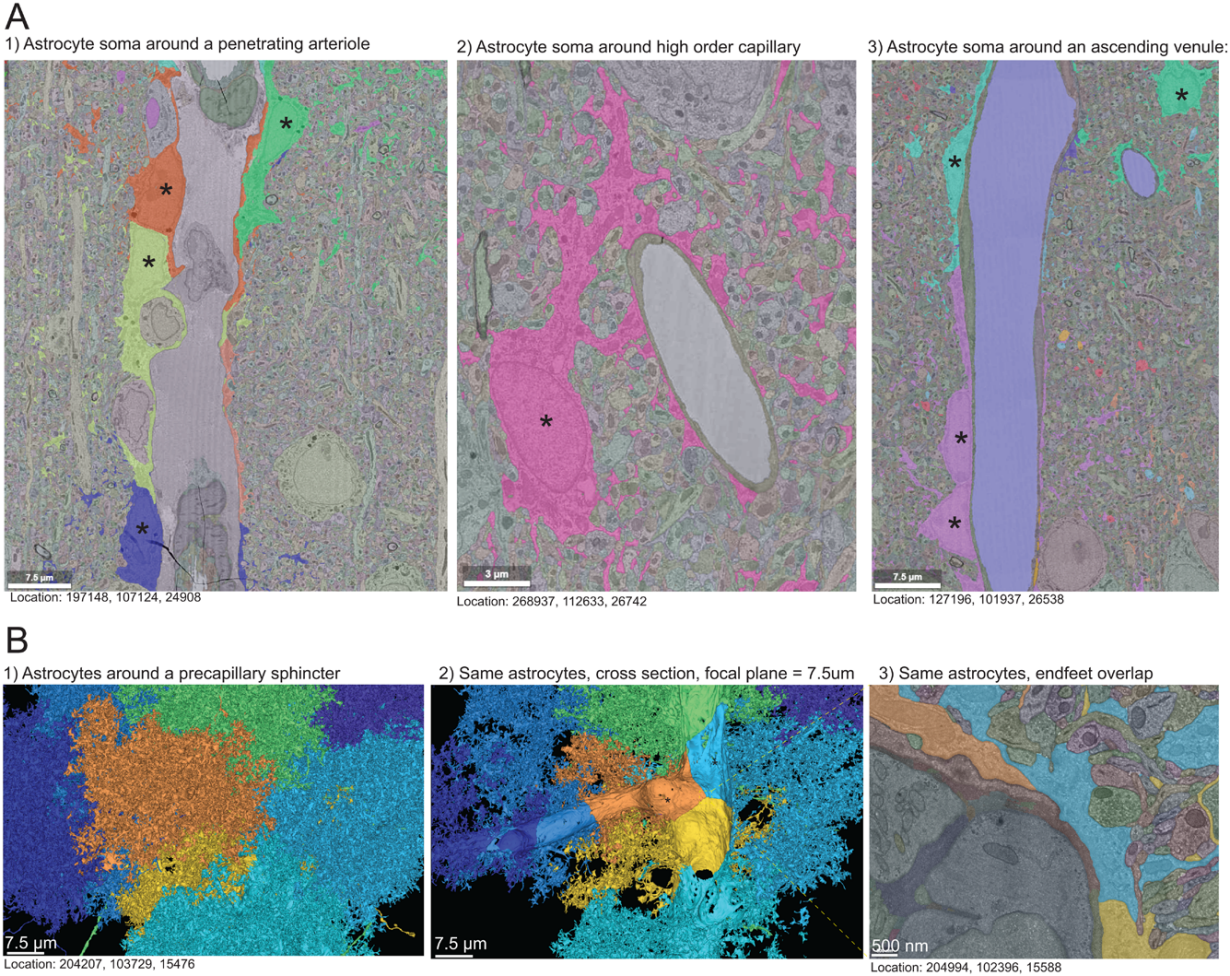
Astrocyte ultrastructure. (A) Ultrastructure shows that astrocyte somas (*) frequently forms the ‘endfeet’ on 1) PAs and 3) ascending venules but the usual depiction of an astrocyte in the parenchyma with endfeet encircling the vessel is typically found on 2) capillaries). (B) 1) 3D segmentation of eight astrocytes around a PA branchpoint shows that the astrocytes each cover a discrete parenchymal volume. 2) By reducing the focal plane to 7.5 µm, the end foot coverage becomes visible and shows discrete coverage with sharp lines where the endfeet meet. Small holes in the center of the endfeet are segmentation errors, not perforations. 3) Ultrastructure shows a large overlap between astrocyte endfeet, without any visible gaps.

These observations support the findings of fluorescence microscopy results (7) which suggest that transitional vascular mural cells in the resistance microvasculature exhibit a continuum of morphologies. Additionally, the precapillary sphincter serves as a boundary for the elastic lamina. While no evident ultrastructural distinctions exist between mural cells encircling precapillary sphincters and those enveloping arterioles, two key features stand out. First, the processes of mural cells overlap at the location of the precapillary sphincter. Secondly, the increased presence of vesicles adjacent to the plasma membrane of mural cells around precapillary sphincters could contribute to the synthesis of the elastic lamina.

### Endothelial cells of first-order capillaries are hotspots for pinocytosis and myoendothelial coupling

Endothelial cells, forming the inner lining of the vasculature in a cobblestone pattern (2), are more elongated at the arterial end than at the venous end. As previously described, at precapillary sphincters the endothelial cell nuclei are often located at the bulbous distention immediately downstream of the precapillary sphincter (Fig. 1C1–4) and are partially uncovered by mural cells (2, 7). Otherwise, most of the arterial-end endothelium is covered by mural cell processes.

**Figure 3:**
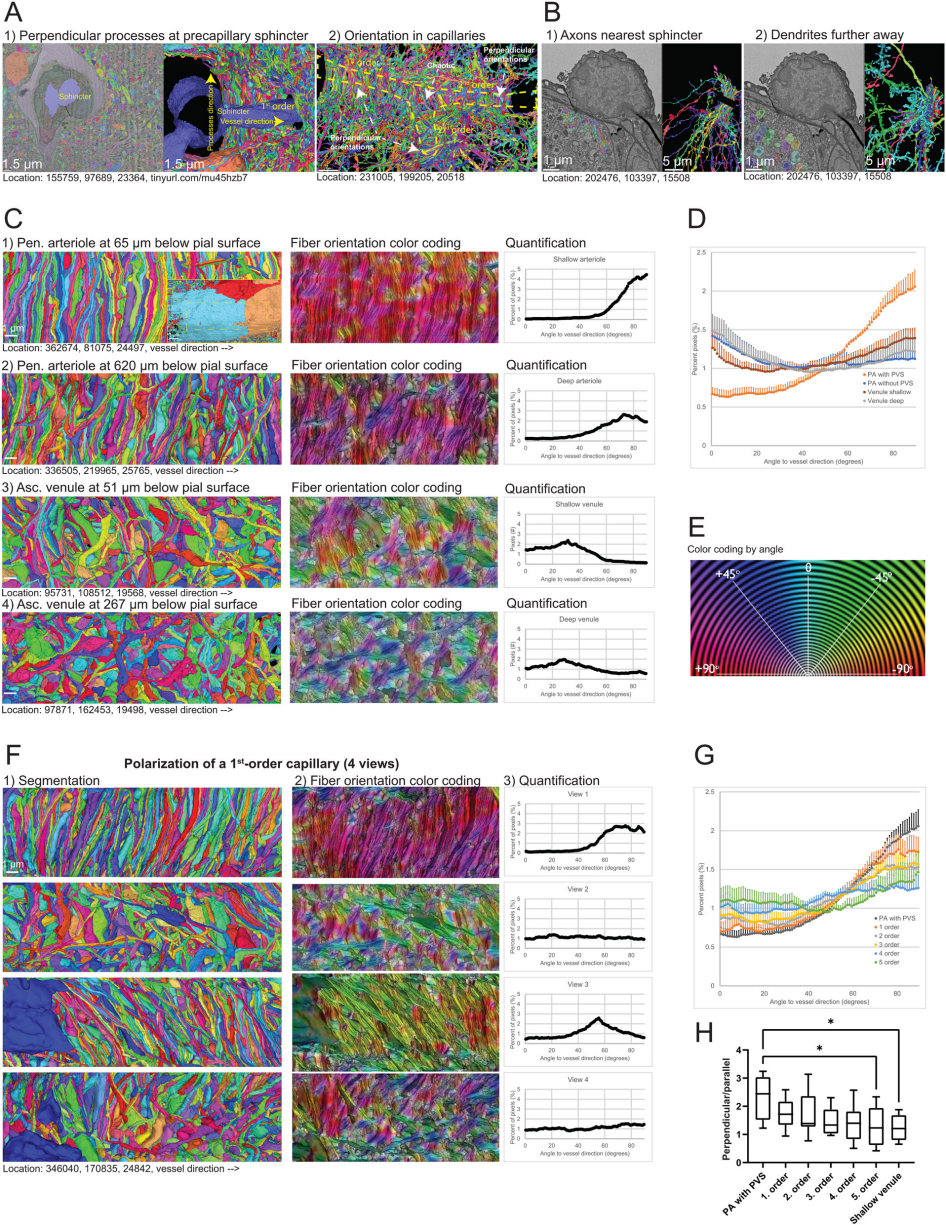
Neuronal processes around the brain microvasculature. (A) 1) Ultrastructure of a PA branchpoint (lumen in blue) where the neuronal processes closest to the astrocytic endfeet are visible in the 3D segmentation (right half). This demonstrates that neuronal processes aligned at a 90° angle to the vessel direction.2) 3D segmentation of neuronal processes around both first- and second-order capillaries. As the vessel bifurcates (dashed yellow line), the perpendicular arrangement is disrupted, but is reestablished after the bifurcation. (B) 1) Ultrastructure and 3D segmentation of axons near the precapillary sphincter. At the precapillary sphincter, the neuronal processes closest to the astrocyte endfeet (A, the endfeet coverage is marked in red) are almost exclusively axons. Dendrites are further away. MC, mural cell; EC, endothelial cell; F, fibroblast. Dashed yellow box shows the approximate location of the EM image. 2)Ultrastructure and 3D segmentation of dendrites near the precapillary sphincter. (C) The perpendicular arrangement is present at PAs, but not at ascending venules. Shown here as segmentation data, color coding of fiber orientation and quantifications of orientation. 1–2) Example of the arrangement of neuronal processes around a PA at 65 and 620 µm below the brain surface. Inset in 1 shows an example of the astrocytic endfeet coverage on top of neuronal processes, which is otherwise removed to show the underlying processes. Yellow dashed line indicates the area that has been analyzed. Holes in endfeet coverage are due to segmentation errors. 3–4)Example of the arrangement of neuronal processes around an ascending venule at 51 and 267 µm below the brain surface. In this example, the neuronal processes are only shown from one viewing angle. Scalebar = 1 µm. (D) Mean percent of pixels of all four views at different angles to vessel direction for PAs with or without PVS (PVS) and for ascending venules shallow or deep in the cortex. Error bars are s.e.m., *n* = 6–7. (E) Indication of angular color-coding of the ImageJ plugin ‘OrientationJ’ that was used to quantify the perpendicular arrangement of neuronal processes. (F) Example of four views, each taken at a 90° angle to each other, of the closest neuronal processes to the astrocytic endfeet (not shown) of a first-order capillary. 1) 3D segmentation of the neuronal processes, the vessel (not shown) is oriented horizontally. 2) Color coding of angles of neuronal processes to the vessel orientation. 3) Quantification of the pixel distribution of angels in the four views. (G) Mean percentage of pixels at different angles to vessel direction for PAs with PVS and first- to fifth-order capillaries. Error bars are s.e.m., *n* = 7–8. (H) Box and whisker plot of ratio between perpendicular (>45°) and aligned (<45°) processes. Tested with one-way ANOVA, *n* = 6–7 per vessel segment. Two outliers were excluded: first order = 4.99 and third order = 4.12.

Endothelial cells can be challenging to visualize in the MICrONS dataset due to segmentation issues; however, luminal protrusions of endothelial cell junctions are discernible as indentations into the vascular lumen segmentation (Supplementary Fig. 1E), enabling identification of endothelial cell borders.

The luminal side of endothelial cells is coated by a layer of glycoproteins (glycocalyx) a component of the tripartite blood-brain barrier (14). The glycocalyx has previously been shown to be nonuniform along the vascular tree, with the highest presence at arterioles and ‘hot spots’ at arteriole branchpoints (15), possibly reflecting the tissue’s shear stress. In the MICrONS dataset, the glycocalyx can be seen as an electron-dense hairy structure, also lining the precapillary sphincter (Fig. 1C4, dark arrowheads).

Rhodin’s description of endothelial cells at precapillary sphincters in the rabbit thigh muscle fascia (8) being 'rich in pinocytic vesicles, particularly on the luminal side' is also reflected in the MICrONS dataset. Endothelial cells at first-order capillaries compared to PAs and downstream capillaries exhibit a significantly higher presence of tubular pinocytic vesicles (Fig. 1C4 and 1C5). Interestingly, first-order capillaries with a precapillary sphincter also have significantly more pinocytic vesicles per area of endothelial cytosol than locations devoid of a sphincter (Fig. 1C5). These resemble findings in the hagfish cerebral endothelium (16) and may play a role in the rapid transcytosis of water and small molecules in a glycocalyx-dependent manner, bypassing lysosomal degradation (17). Supporting this, luminal membrane ruffles in these endothelial cells resemble micropinocytic invaginations (Fig. 1C4, Supplementary Video 2).

Myoendothelial junctions are locations where the endothelial cells and mural cells reach across the basal lamina or elastic lamina to touch and connect via gap junctions. Rhodin found that myoendothelial junctions, in precapillary sphincters were ‘more pronounced’ and ‘frequent’ compared with arterioles, with approximately 10–20% of the precapillary-sphincter basal endothelial plasma membrane specialized as myoendothelial junctions (8). Consistent with this, in the MICrONS dataset, myoendothelial junctions are significantly more frequent at proximal first-order capillaries compared with PAs or second-order capillaries (Fig. 1D1–3 and Supplementary Video 3). However, this characteristic is not exclusive to precapillary sphincters. The high concentrations of myoendothelial junctions are similarly observed in non-sphincter first-order capillaries (Fig. 1D3). Interestingly, myoendothelial connections (’peg-and-sockets’) are also abundant at higher-order capillaries where they may have a role in adhering thin-strand pericytes to the endothelium (18). Myoendothelial junctions are enriched along the edges of mural cell processes. Contrary to Ornelas *et al.* (18), who looked at ‘pegs-and-sockets’ near an ascending venule, in the arterial end they are not particularly enriched near the mural cell somas.

These findings suggest that first-order capillaries are hotspots for myoendothelial communication and that the presence of a precapillary sphincter increases the rapid transcytosis of water and small molecules across the endothelium.

### Perivascular fibroblasts reinforce the resistance microvasculature

The resistance microvasculature, which experiences the highest blood pressure within the microvascular system, plays a crucial role in maintaining vascular stability. Mural cells inherently have a tonus to counteract this pressure, but there is a secondary line of defense: collagen fibers. Produced by fibroblasts (19), these fibers not only reinforce the vasculature but also limit its dilation when the tonus is fully relaxed.

Perivascular fibroblasts, distinct from pericytes and potentially a continuation of pial fibroblasts (20), can be identified by their long slender processes and their location, which is abluminal to mural cells and luminal to astrocytic endfeet and/or macrophages. Unlike macrophages, which I have described in another study (21), they contain only a few lysosomes.

Collagen fibrils are visible in the MICrONS dataset as thin parallel fibrils with a diameter of approximately 50–100 nm with cross-striations (Fig. 1C6, E and G, Supplementary Fig. 2A and C, Supplementary Video 4). Invaginating pockets (fibripositors) with terminal collagen fibrils can be found on the luminal side of fibroblasts (Supplementary Fig. 2D), indicating collagen fibril assembly (Supplementary Fig. 2D and Supplementary Video 4).

**Figure 4:**
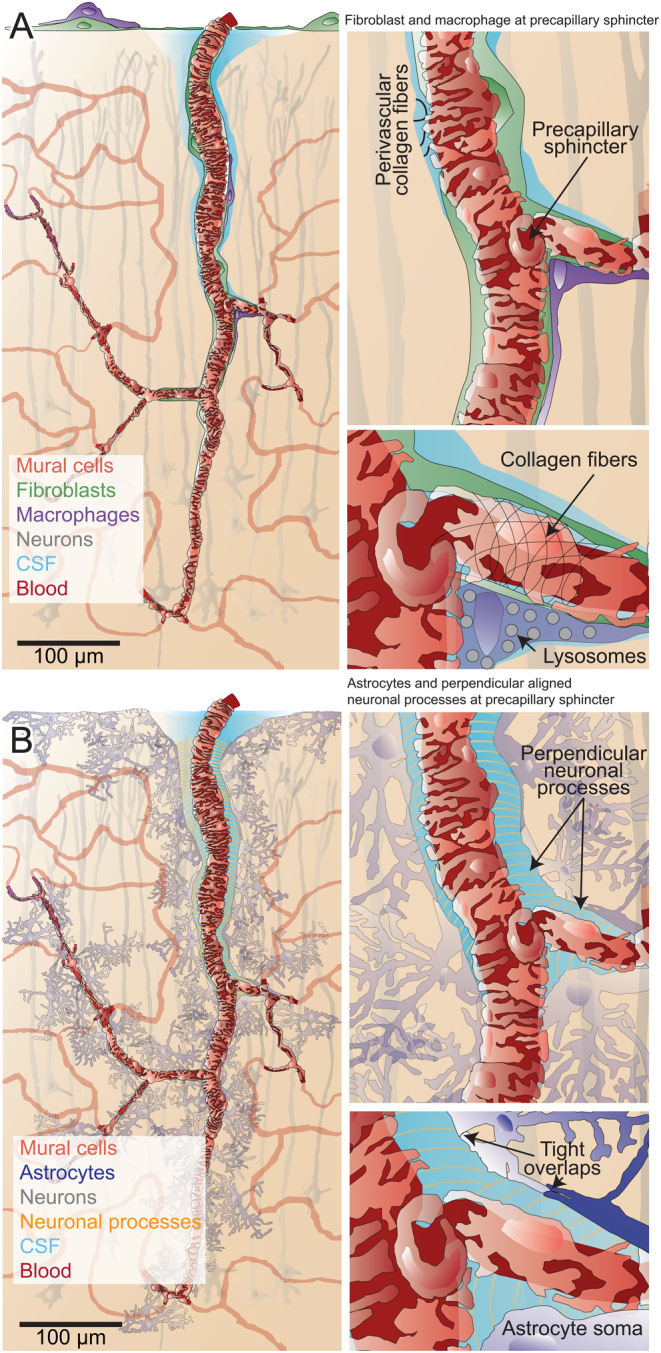
Illustrations of the neurovascular unit of the resistance microvasculature. (A) Left panel: Illustration of a PA branching to a precapillary sphincter. The depicted cell types include mural cells, neurons, perivascular fibroblasts and macrophages, pial fibroblasts, and a meningeal macrophage. Right panel: A magnified view of the precapillary sphincter branchpoint, demonstrating how fibroblast- and macrophage processes reach the first-order capillary. (B) Left panel: This illustration depicts how astrocytic endfeet envelop the entire PVS and glia limitans. Note the inclusion of astrocyte cell bodies as part of the endfeet on the PA. Right panel: A magnified view of the precapillary sphincter branchpoint, showing how neuronal processes aligned perpendicular to vessel direction exist both on the PAs where there is a PVS and on arterial-end capillaries that may also have a PVS.

An example of a perivascular fibroblast next to a PA-branchpoint mural cell has been observed previously in the MICrONS dataset (11). However, the cytoarchitecture of that cell included electron dense lysosomes and phagosomes, suggesting that it is a macrophage, not a fibroblast. In contrast, a fibroblast is found on the luminal side of that macrophage (Supplementary Fig. 2E). Perivascular fibroblast somas are found on pial arterioles and PAs, ascending venules and on first-order capillaries but rarely beyond that point (Fig. 1C3–4 and Fig. 4A), with processes reaching third-order capillaries. Where the fibroblast processes exist, collagen fibrils can also be found (Fig. 1C6).

A fibroblast soma and/or macrophage soma location next to the precapillary sphincter mural cell is often associated with an indentation in the PA lumen (Supplementary Fig. 2F), a phenomenon also observed *in vivo* (7).

Perivascular spaces (PVS) can be found around PAs and are occasionally 3D segmented (Fig. 1F). Collagen fibrils are also present at the luminal side of the astrocytic endfeet, visible in presence of a PVS (Fig. 1G). Some collagen fibrils even connect the abluminal mural cell basement membrane with the astroglial basement membrane. Fibroblasts are only in direct contact with the endothelial basement membrane in places devoid of mural cells (Supplementary Fig. 2G).

These findings illustrate how fibroblasts synthesize collagen fibrils to fortify the resistance microvasculature and how the fibrils extend across the PVS to connect mural cells with astrocytic endfeet.

### Astrocytic endfeet overlap to form a barrier

Astrocytes are typically depicted as a star-shaped cell body situated in the parenchyma with long processes that form endfeet around blood vessels. They can be identified in the 3D segmentation by their endfeet covering the vasculature and the many fine processes encircling neuronal synapses. In a recent review, we proposed that astrocytes may have their soma located directly at blood vessels (2). In the MICrONS dataset, it appears that for large vessels (both arterioles and venules), this is more the norm than the exception (Fig. 2A1, Fig. 2A3, Fig. 4B and Supplementary Video 5), whereas, for capillaries, the astrocyte soma is often positioned in the parenchyma (Fig. 2A2). From the astrocyte soma, large processes extend out in the parenchyma and divide into smaller processes that come in close contact with neuronal synapses.

Astrocytes have well-defined domains that are populated by the fine processes and endfeet (Fig. 2B). The astrocytic endfeet overlap (Fig. 2B1–3) and contribute to the blood–brain barrier’s restrictive function by limiting diffusion (22). Occasionally, openings between astrocytic endfeet can be found where a single axonal bouton protrudes to contact a blood vessel, but I have only observed that in high-order capillaries (Supplementary Fig. 3A). Notably, some endfeet become extremely thin, giving the false impression of incomplete coverage (Fig. 2B2). A close inspection of the EM data reveals that segmentation artifacts caused by the collapsed end foot plasma membrane blends in with the extracellular matrix of similar electron density.

Collectively, these examples demonstrate that astrocyte soma form a part of the perivascular glia limitans of PAs and ascending venules in addition to endfeet coverage, and that astrocytic endfeet form a tightly overlapping barrier which may limit paracellular communication.

### Brain precapillary sphincters are not directly innervated

Perivascular ‘extrinsic’ nerve fibers, originating from the sympathetic nervous system, innervate cerebrovascular arteries, and it is believed that they exist on cortical vessels up until their entry into the brain parenchyma (23), including the resistance microvasculature (24). However, I have not found any ultrastructural evidence of this in the literature, besides a study by Krimer *et al.* (25) showing electron-dense smudges on the parenchymal side of a couple of capillaries, which they claim are ‘giant dopaminergic boutons’. Unfortunately, from the quality of their images, it is not possible to assess whether these structures are parenchymal or perivascular or whether they are indeed boutons. Rhodin observed ‘abundant’ unmyelinated nerve endings near the precapillary sphincter in the rabbit thigh muscle fascia, with axonal-terminal boutons containing vesicles with granules (8), suggesting direct neuronal modulation of precapillary-sphincter function in the peripheral tissue. However, in the MICrONS dataset, I found no perivascular ‘extrinsic’ innervation of cortical PAs, precapillary sphincters (Fig. 3A1) or even of the largest pial arterioles or venules (Supplementary Fig. 3B1–2).

Interestingly, unmyelinated axonal processes are abundant closest to the astrocytic endfeet of PAs and first-order capillaries, with synaptic vesicle-filled boutons facing the endfeet, while dendrites and dendritic spines most often lie in the second row (Fig. 3B and Supplementary Video 2). At high-order capillaries, there are comparatively more dendrites closest to the vasculature. Unfortunately, many segmentations of axonal processes are incomplete in the MICrONS dataset, making it challenging to characterize the origin of these processes. However, of the neuronal processes near the first-order capillaries where the origin can be traced, one can find: spiny dendrites of pyramidal cells as well as processes and somas of many types of interneurons, including Martinotti cells, chandelier cells, and oligodendrocyte precursor cells (Supplementary Fig. 3C). Essentially, any type of neuron one might expect in the cortex can be found in this region.

These findings demonstrate that, contrary to peripheral microvasculature, brain precapillary sphincters are not innervated by perivascular ‘extrinsic’ nerve fibers. With a few exemptions at high-order capillaries (Supplementary Fig. 3A), all neuronal processes I have observed reside at the parenchymal side of astrocytic endfeet.

### Neuronal processes are aligned perpendicular to the vascular direction

While examining the NVU ultrastructure in the MICrONS dataset, it became evident that ‘intrinsic’ parenchymal neuronal processes closest to astrocytic endfeet at PAs and first-order capillaries exhibit a surprising organization.

These neuronal processes arrange themselves perpendicular to the vessel direction and curve around the vessel, particularly near the precapillary sphincter (Fig. 3A, Fig. 4B and Supplementary Video 6). This arrangement appears to be independent of parenchymal orientation or vessel depth, as it can be observed both at PAs and capillaries branching at different angles (Fig. 3A), both shallow and deep in the cortex (Fig. 3C). At capillary bifurcations, neuronal processes appear more chaotic, but the organization is reestablished on daughter branches (Fig. 3A). Interestingly, this perpendicular organization seems to be limited to one half of the perivascular wall, while the second half often has one or more cell bodies (or thicker astrocytic endfeet) occupying space (Supplementary Fig. 4A). These can be astrocyte cell bodies (Supplementary Fig. 4A1), microglia (Supplementary Fig. 4A2), neuronal cell bodies (Supplementary Fig. 4A3–4), or oligodendrocyte precursor cells (Supplementary Fig. 4A4). Interestingly, the cell bodies occupying space are often abnormally elongated (Supplementary Fig. 4A2–4). Occasionally, in the space between the two halves of the perivascular wall, neuronal processes can be seen following the vessel direction (Fig. 3F, views 2 and 4).

Synapses are present throughout the parenchyma and can therefore also be found near the arterioles and first-order capillaries (Fig. 1C4); however, axonal boutons seem more concentrated on the second half of the perivascular wall further away from the vasculature (Fig. 3F, views 2 and 4, and Supplementary Video 6).

To quantify the arrangement of neuronal processes closest to the astrocytic endfeet, their orientation was analyzed by averaging four orthogonal views of each segment. This was done for PAs and first- to fifth-order capillaries, as well as ascending venules. If the processes follow the vessel direction, their angles approximate 0° and if they are perpendicular, their angles are close to 90°. The majority of the capillary trees examined feature approximately eight capillary bifurcations between the PA and ascending venule when considering the shortest distance. Notably, I did not identify any capillary trees that connected PAs with ascending venules with fewer than five bifurcations. This observation underpins the rationale for including five capillary orders in the analysis.

Parenchymal neuronal processes near the astrocytic endfeet of PAs with PVSs displayed a significantly higher incidence of neuronal processes going perpendicular to the vessel direction (Fig. 3C1 and D). Interestingly, PAs without PVSs, typically found deeper in the cortex and possessing smaller lumen diameters, did not exhibit this (Fig. 3D). In a particular instance, a large PA had a PVS reaching 489 µm and maintained a substantial diameter to this depth due to lack of bifurcations; it also had neuronal processes arranged perpendicular to vessel direction, even deep in the cortex (Fig. 3C2, Supplementary Video 6). This observation challenges the notion that the perpendicular arrangement is merely a result of horizontally oriented neuronal processes cortex’s molecular layer. Supporting this, venules located either near the surface or deep in the cortex do not exhibit many neuronal processes perpendicular with the vessel direction (Fig. 3C3–4 and D), and venules in the MICrONS dataset do not possess any PVSs, except for the initial few micrometers below the pial surface (21).

In line with the differences between arterioles and venules, the perpendicular arrangement diminishes with capillary bifurcations (Fig. 3G and H), and by the fifth-order capillaries, the processes are significantly less perpendicularly rectified than at PAs with PVS.

These striking findings illustrate how neuronal processes nearest to the astrocytic endfeet on one half of the vascular wall are arranged perpendicular to the vessel direction, not only at PAs with PVS but also at arterial-end capillaries.

### Perpendicular arrangement is also found in human cortical vasculature

In the ‘H01-release’ human cortex dataset (10), also available via Neuroglancer, a similar arrangement of neuronal processes half-circling the parenchymal perivascular wall is observed (Supplementary Fig. 4D and Supplementary Video 7), suggesting that this arrangement is not exclusive to mice or an artifact of the MICrONS sample.

The segmentation in the H01-release dataset is much more fragmented and less accurate, and astrocytic endfeet appear severely swollen, complicating the identification of vascular cells (11). However, based on the presence of contractile elements in the mural cells (Supplementary Fig. 4B), a PA with a first-order-capillary branchpoint could be discerned. The 3D segmentation reveals perpendicular processes on one side of the first-order capillary, and on the opposite side, six oligodendrocyte cell bodies are arranged back-to-back (Supplementary Fig. 4C, tinyurl.com/2yc3znte). Between the two sides, neuronal processes align with the vessel direction (Supplementary Fig. 4D, Supplementary Video 7).

Segmentation of a human ascending venule did not show any alignment of neuronal processes (Supplementary Fig. 4D, Supplementary Video 7), confirming that the polarization and perpendicular arrangement of neuronal processes are specific for the arteriolar side and are conserved between mice and humans.

These findings suggest that the arrangement of neuronal processes perpendicular to vessel direction observed in the arterial end of human cortical vasculature is conserved, indicating the translatability from mouse to human; however, this needs to be confirmed when more public large volume EM datasets of the human brain exist.

## Discussion

### Transitional mural cells exhibit a continuum of morphologies

Mural cells confer tonus to blood vessels, a fundamental characteristic for the regulation of blood flow. As demonstrated in this study and in previous work (2, 7), mural cells on PAs and first-order capillaries present a continuum of morphologies (Fig. 1A, B, C and Fig. 4A). Therefore, it is an oversimplification to categorize PA mural cells solely as smooth muscle cells. Moreover, it contradicts Zimmermann’s original definition of pericytes, which includes all transitional forms from spindle-shaped smooth-muscle cells (not included) to capillary thin-strand pericytes (2, 26), suggesting that they should all be referred to as pericytes.

In the MICrONS dataset, contractile elements can be observed in mural cells of arterioles and first- to fourth-order capillaries (Supplementary Fig. 1A), but not beyond this point. While contractility has been proposed for pericytes beyond the fourth-order capillary (27, 28) (thin strand pericytes), this is a very slow process thought to involve cytoskeletal elements like g- or f-actin. However, this theory largely rests on questionable α-SMA labeling in a highly referenced study (29) and confusing differences in capillary-order nomenclature between retina and brain (30). Hence, I propose to refer to the transitional mural cells as either ‘contractile mural cells’ or ‘noncontractile mural cells’, depending on their functional characteristics.

### The ultrastructure of precapillary sphincters

Precapillary sphincters, an often-overlooked feature of cerebral microvasculature (31), have been brought to light through our recent descriptions of their presence and function using *in vivo* two-photon imaging and immunohistochemistry (2, 7). The MICrONS dataset offers a unique opportunity to explore the ultrastructure of precapillary sphincters within the context of cortical depth and microvascular hierarchy.

A previous study utilizing the MICrONS dataset (11) has illustrated a PA branchpoint encircled by mural cells (Supplementary Fig. 2E). However, despite the presence of a mural cell soma with encircling processes at this branchpoint, it does not meet our definition (7) of a precapillary sphincter due to the absence of lumen indentation at the branchpoint (Supplementary Table 1, arteriole 2 branch 1).

The contractile mural cells encircling precapillary sphincters and the overlapping mural cell processes at the branchpoint indentation are consistent with the high contractility of precapillary sphincters (7, 32), reinforcing the idea that these structures play a pivotal role in blood-flow regulation. In the following sections, I will delve into the main findings from the ultrastructure of precapillary sphincters.

### The precapillary sphincter may be important for transcytosis and fluid uptake

The precapillary sphincter, a site of significant fluctuations in blood pressure and hydrostatic pressure (7), is posited to be a critical zone for fluid filtration from the blood to the PVS. This filtration typically occurs paracellularly in most tissues. However, in the brain, the presence of endothelial tight junctions restricts paracellular transport, suggesting that transcytosis may play a crucial role in fluid uptake from the blood to the PVS. Under normal conditions, transcytosis is suppressed in brain endothelial cells, with exceptions observed in disease states (33). A high number of vesicles could be indicative of cellular damage, such as during the fixation process. However, the predominance of tubular micropinocytic vesicles in the precapillary sphincter endothelium (8) (Fig. 1C4–5), coupled with the general absence of other signs of cellular damage like swollen mitochondria or astrocytic endfeet, suggests that the abundance of these vesicles is more likely related to their location rather than being an artifact. Supporting this notion, tubular vesicles in brain endothelial cells are hypothesized to participate in rapid transcytosis, a process facilitated by PACSIN-2 (17). Additionally, abrupt changes in the composition of the glycocalyx (Fig. 1C4) may influence transcytosis (17). The luminal membrane ruffles observed in first-order capillary endothelial cells (Fig. 1C4, Supplementary Video 2) further suggest that transcytosis is directed from the blood towards the brain, aligning with the requirement for fluid uptake.

### Myoendothelial junctions are more abundant at the precapillary sphincter

Johannes Rhodin speculated whether myoendothelial junctions were more than just a structural component that stabilize the microvascular wall (8). These junctions are sites where contractile mural cells connect with endothelial cells via gap junctions (34). The hyperpolarizing signal in NVC, conveyed by inward rectifier K^+^ channels in endothelial cells, may propagate through these myoendothelial gap junctions to contractile mural cells, thereby inducing vasodilation (35, 36, 37). The high concentration of myoendothelial junctions spanning the elastic lamina at the precapillary sphincter (Fig. 1D1–3) could account for the early response of proximal first-order capillaries to nerve activity (38).

Myoendothelial junctions are primarily located along the edges of mural cell processes (Supplementary Video 3) and can be protrusions from endothelial cells or mural cells or both. The presence and type of connexins in all these myoendothelial junctions, and whether they contain gap junctions, remains uncertain (39). Some of these junctions may simply serve as ‘peg-and-socket’ attachment points (18). However, the first-order capillaries are expected to have a strong myoendothelial coupling (38, 40), and the abundance of myoendothelial junctions at precapillary sphincters suggest that these are crucial sites for NVC.

### The elastic lamina ends at precapillary sphincters

The ultrastructure of elastin can be observed in arterioles in the MICrONS dataset, confirming the termination of elastic lamina at the precapillary sphincter (Fig. 1E and Supplementary Fig. 2B), as previously reported (7). This contrasts with Rhodin’s conclusion that no elastic components are present in relation to the precapillary sphincter ultrastructure in the rabbit thigh muscle fascia (8). This discrepancy may suggest a differential need for microvascular elasticity between the brain and other tissues, potentially due to differences in the need to dampen pulse-pressure waves, given that the thigh muscle fascia is less perfused and more distal to the heart compared to the brain.

Interestingly, the presence of elastin in arterioles but not capillaries could provide an explanation for the functional presence of K_ir_2.1 in arterioles but not capillaries of mice with cerebral autosomal dominant arteriopathy with subcortical infarcts and leukoencephalopathy (CADASIL) (41). TIMP3, an inhibitor of K_ir_2.1 channel activity that accumulates in CADASIL mice, binds with high affinity to elastin, potentially shielding the endothelium from TIMP3 exposure (41, 42). It would be intriguing to investigate whether differences in the expression of elastin in brain and peripheral microvasculature could influence the outcomes of CADASIL.

### Perivascular collagen fibrils may couple arteriole constriction with astrocyte activity

While the coverage of elastin concludes at the precapillary sphincter, fibroblast processes continue to enwrap the first-order capillaries, with collagen fibrils also present (Fig. 1C6, Supplementary Video 2), providing structural support. These fibrils may also restrict the maximum dilation of capillaries (7). On PAs, collagen fibrils that extend to the astroglial basement membrane across the PVS (Fig. 1G) may play a role in activating mechanosensitive membrane proteins such as TRPV4. This could lead to an increase in calcium in astrocytic endfeet, triggering the release of vasodilators as part of a stretch-mediated feedback mechanism, resulting in oscillations of arteriole diameter (43). Conversely, a recent study suggested that macrophages degrade these PVS-crossing collagen fibrils and demonstrated that depletion of macrophages limits arterial motion (44).

### Neurovascular coupling is astrocyte dependent

NVC encompasses several pathways, including nitric oxide-, glutamate- and purinergic-signaling pathways (2, 45). These can be categorized into gasotransmitters (such as NO, CO, and H_2_S), neurotransmitters (such as glutamate and acetylcholine) and neuromodulators (such as ATP). These pathways involve intracellular calcium signaling and changes in membrane ion conductance or electrochemical gradients.

It is questionable whether NVC occurs paracellularly in between the tightly overlapping astrocytic endfeet (Fig. 2B), suggesting that neuronal processes either signal through astrocytic endfeet (4, 5) or via gasotransmitters that can pass through the endfeet unhindered.

Nitric oxide (NO) is hydrophobic and highly diffusible and is thought to be the primary facilitator of vasodilation (46). However, in the cerebral cortex, NO serves as a modulator (playing a permissive role) rather than a direct mediator of NVC (47). Pathways involving astrocytic endfeet signaling are considered the most likely mediators of NVC, particularly for capillaries (4).

Astrocyte-dependent pathways involve intracellular calcium signaling, which can lead to either a vascular dilation or constriction, depending on the levels of NO and brain metabolic elements (2, 48). However, only about a third of NVC can be explained by known mechanisms, suggesting the potential involvement of yet unidentified mechanisms (46).

### Perpendicularly aligned neuronal processes may enhance neurovascular coupling

The discovery that parenchymal nerve processes align perpendicularly to the direction of blood vessels at the arterial end where a PVS is present (Fig. 3 and Supplementary Fig. 4), as well as at the contractile first- to fourth-order capillaries where a PVS is generally absent, prompts the question of whether this arrangement plays a role in enhancing NVC?

Neuronal processes aligned perpendicularly could potentially (i) allow a greater number of processes to be in proximity to the vessel; (ii) minimize the extracellular space between them (compared with a more chaotic arrangement), thereby locally concentrate the extracellular K^+^ released by action potentials, leading to increased K^+^ siphoning by astrocytes (although astrocytic K^+^ siphoning is not likely a major mechanism of NVC (49)); (iii) minimize the distance that membrane-permeable molecules like NO, O_2_, or CO_2_ need to diffuse to reach their targets; (iv) minimize the distance of signaling through the astrocytic endfeet; (v) lead to better sense and react to changes in blood flow (the hemoneural hypothesis (3)). Moreover, the observed polarization of perpendicular alignment suggests that synapses (and therefore neurotransmitter release) are concentrated in certain areas near the vessel.

While these suggestions could explain the advantages of the alignment of neuronal processes, they do not account for why such alignment is predominantly found around PAs with a PVS, although the increased diffusion distance across the PVS might make it more significant. The cause of these perpendicular alignments is also unclear. They could be influenced by vascular wall signaling during the migration of the neuronal processes, controlled by astrocyte processes, or affected by perivascular pumping. Alternatively, nonaligned processes could be pruned by microglia. Regardless the cause and mechanism (if any), further research is needed to clarify this.

To summarize, the ultrastructure of the NVU reveals that (i) the precapillary sphincter is central to the regulation of blood flow and transcytosis; (ii) the barrier function of astrocytic endfeet has likely been underestimated; (iii) perpendicularly aligned neuronal processes encircle PAs and first- to fourth-order capillaries which may enhance NVC.

## Conclusion

Capillaries can be stimulated through a variety of mechanisms, including chemical signaling pathways that involve neurotransmitters and gasotransmitters, electrical signals arising from changes in ion conductance and electrochemical gradients across the plasma membrane, and mechanical signals generated by the stretching of vascular mechanosensitive ion channels and receptors. Precapillary sphincters and first-order capillaries of the resistance microvasculature play a pivotal role in this communication. In the brain, neuronal synapses exist within hundreds of nanometers of the resistance microvasculature; however, they are separated by nonfenestrated astrocytic endfeet. Neurovascular coupling must therefore be mediated by transcellular and/or paracellular signaling through tightly overlapping astrocytic endfeet. The efficiency of this signaling could be enhanced by the perpendicular alignment of neuronal processes to the direction of the blood vessels, concentrating signaling ions and molecules and decreasing their diffusion distances.

## Limitations

While the MICrONS dataset does exhibit some instances of astrocyte swelling, the extent is relatively limited. However, in the human dataset (h01), the astrocytic endfeet are significantly swollen, which complicates the identification of arterioles and capillary orders, and poses challenges for the characterization of the NVU. Additionally, the process of chemical fixation may lead to a reduction in the extracellular space and potentially also the PVS, further complicating the analysis. However, the MICrONS dataset and the h01 dataset are unique in the sense that they are the first publicly available large 3D ultrastructural datasets of a size that enables you to follow the vascular hierarchy. Hopefully more datasets of this magnitude will be produced.

## Supplementary Materials

Supplementary Figure 1

Supplementary Figure 2

Supplementary Figure 3

Supplementary Figure 4

Supplementary Table 1 – quantification of precapillary sphincters:

Video 1. Showing the morphology of mural cells (various colors) encircling a PA, precapillary sphincter and 1st - 3rd-order capillaries. The vascular lumen is light blue and endothelial cells are not shown. Notice how the morphology transitions from mural cells with a more flat soma and 3-4 processes encircling the PA (red, purple and cyan), while the ensheathing pericyte at the precapillary sphincter (cyan) and the ensheathing pericyte covering the 1st- and 2nd-order capillaries (pink) have more protruding somas and several processes ensheathing the capillaries. The thin-strand pericyte on the 3rd-order capillary also have a protruding soma, but the processes are increasinglu oriented along the capillary rather than encircling it. The location in the MICrONS dataset is: 225010, 158639, 19156. N.B. the cyan segmentation contains both an ensheathing pericyte at the precapillary sphincter, parts of a PA mural cell and parts of a neuronal process.

Video 2. Showing individual layers in the MICrONS dataset serial EM of the PA and precapillary sphincter shown in Figure 1c. The video is paused at different layers to show the presence of collagen fibers at the bulb (part of 1st-order capillary), the location of endothelial cells, mural cells, fibroblasts, a macrophage and astrocytes, contractile elements, differences in glycocalyx density, pinocytic vesicles, elastin, myoendothelial junctions and junctions between mural cells (myo-myo junctions). The location in the MICrONS dataset is: 203779, 103491, 15475.

Video 3. Showing annotations (yellow dots) of myoendothelial junctions and/or peg-and-socket junctions at a PA, a precapillary sphincter, a 1st order capillary, 2nd- and 3rd-order capillaries. Mural cells (various colors) and vascular lumen (light blue) have a low opacity to make the annotations visible. Mural cell somas are marked by yellow asterisks. Notice how relative to the PA and 1st-order capillary the precapillary sphincter has many myoendothelial junctions. This is also depicted in Figure 1d and the location in the MICrONS dataset is: 349084, 92330, 25337.

Video 4. Showing individual layers in the MICrONS dataset serial EM of a vascular wall of a PA where endothelial cells, a mural cells, a fibroblast, the PVS and astrocytic endfeet are indicated. The video pauses at a location where pockets in the fibroblasts contains leading end of collagen strands (fibrils); however, this can be appreciated best by playing and rewinding the movie while following the collagen fibers. Collagen fibers can also be seen traversing the PVS. This is also depicted in Supp. Fig. 2d and the location in the MICrONS dataset is: 380203, 87715, 27397.

Video 5. Showing individual layers in the MICrONS dataset serial EM while moving to follow the astrocyte endfeet around a PA with PVS. The astrocyte segmentations are in various colors, but the green astrocyte is mistakenly segmented together with mural cells. Not all astrocyte segmentations are shown. Notice how the astrocyte somas form a part of the endfeet and how the astrocytic endfeet overlap substantially. This is also depicted in Figure 2a and the location in the MICrONS dataset is: 201978, 82707, 24442.

Video 6. Showing 3D segmentations of mouse neuronal processes (various colors) on the abluminal side of astrocytic endfeet for a shallow PA and a shallow 1st order capillary with PVS, a deep PA without PVS, a shallow venule without PVS and a deep PA with PVS. The vasculature and PVS are not shown, but the vascular direction is indicated (horizontally). Each movie starts with cross-sectional view, increases the focal depth, turns to a side-view and tilts.

Video 7. Showing 3D segmentations of human neuronal processes (various colors) on the abluminal side of astrocytic endfeet for a 1st order capillary (Location in the H01 dataset: 277102, 180552, 5259) and a venule (Location in the H01 dataset: 353116, 70815, 3916). The vasculature is not shown, but the vascular direction is indicated (horizontally). Each movie starts with cross-sectional view, increases the focal depth, turns to a side-view and tilts.

## Declaration of interest

The author declares that there is no conflict of interest that could be perceived as prejudicing the impartiality of the study reported.

## Funding

This study did not receive any specific grant from any funding agency in the public, commercial, or not-for-profit sector.
