# Supplementary figures and images for: Ultrastructure of precapillary sphincters and the neurovascular unit
Source: Vasc Biol. 2023 Dec 1;5(1):e230011. doi: 10.1530/VB-23-0011 (PMC10762554; doi:10.1530/VB-23-0011)

# Supplementary Figure 1

a)

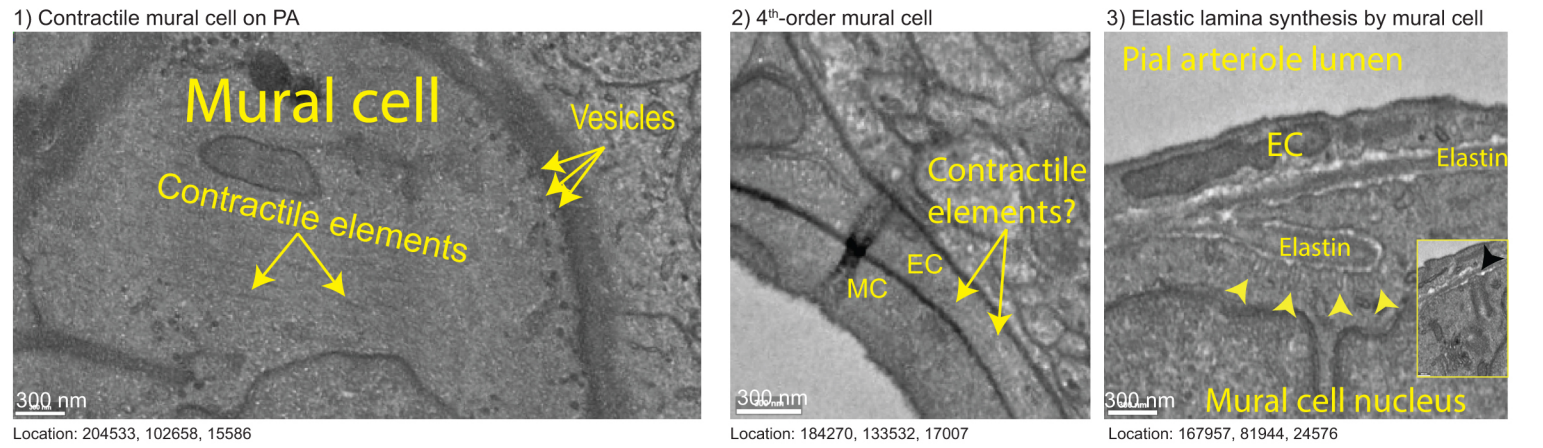

b)

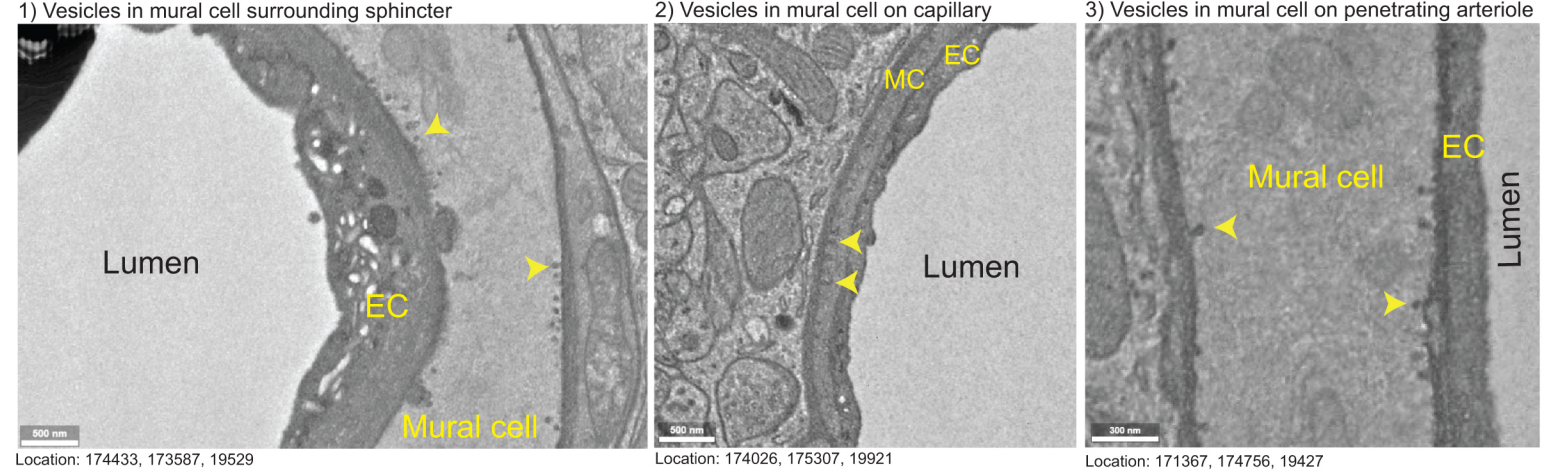

c)

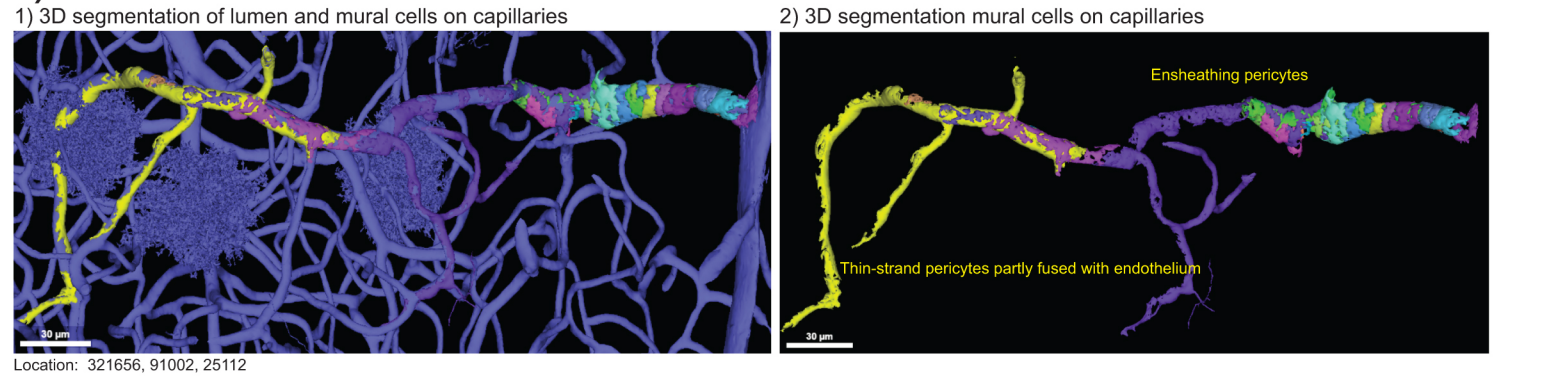

d)

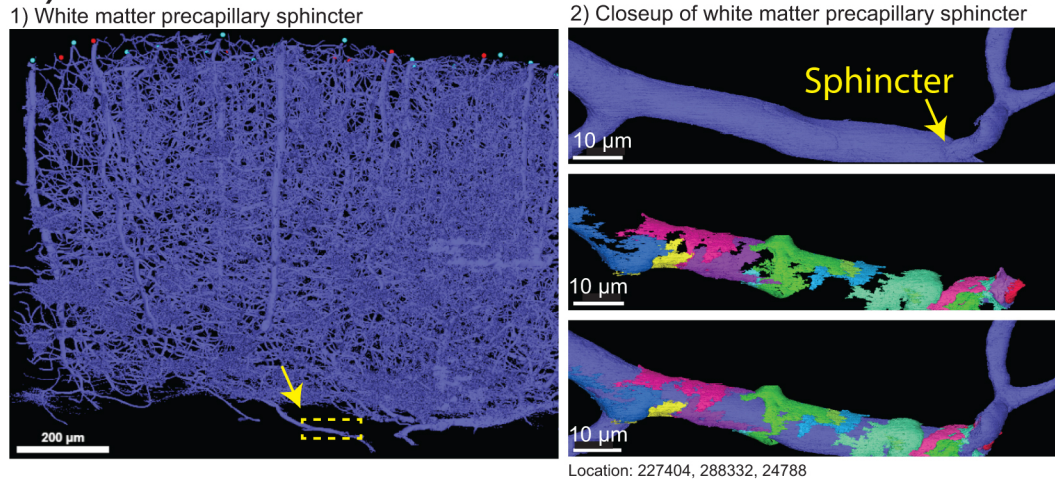

e)

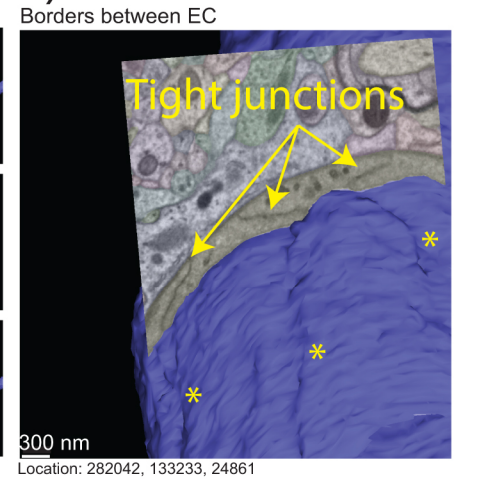

Supplement: Supplementary Figure 1 [file supplementary_figure_1.pdf]
