# Supplementary material for: Ultrastructure of precapillary sphincters and the neurovascular unit
Source: Vasc Biol. 2023 Dec 1;5(1):e230011. doi: 10.1530/VB-23-0011 (PMC10762554; doi:10.1530/VB-23-0011)

Supplementary Figure 3

a) 1) Axonal bouton plugs a hole between astrocytic endfeet

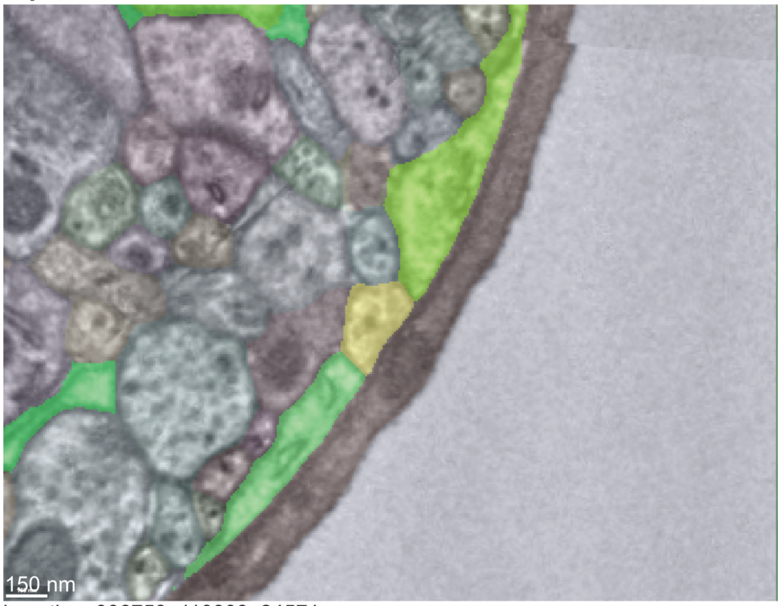

2) 3D segmentation of plug

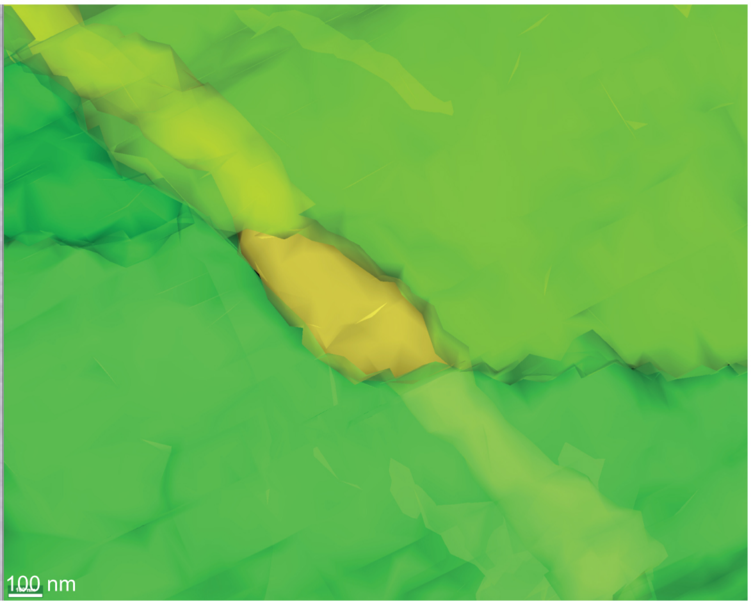

Location: 306753, 110808, 24571

b)

1) Large pial arteriole with no perivascular nerve processes

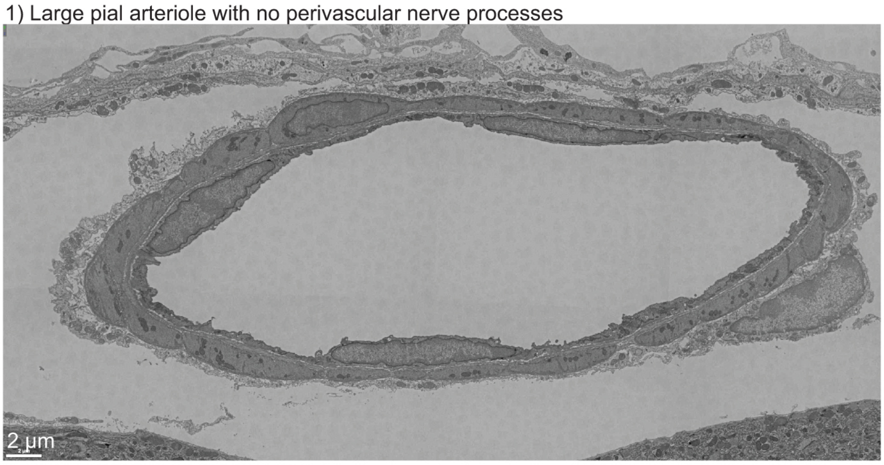

Location: 255636, 81237, 14829

2) Large pial venule with no perivascular nerve processes

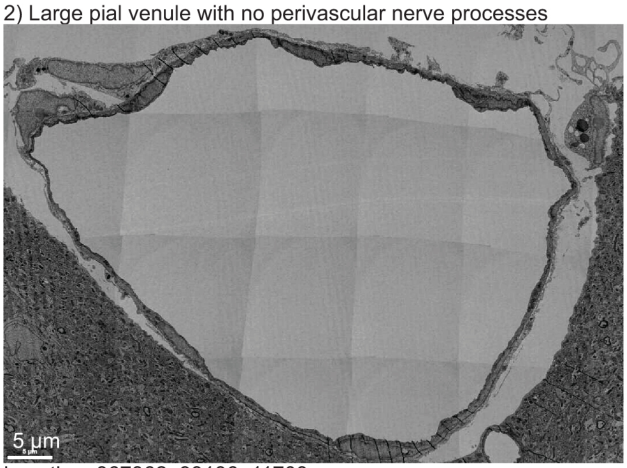

Location: 367362, 93136, 11703

c)

Pyramidal cells

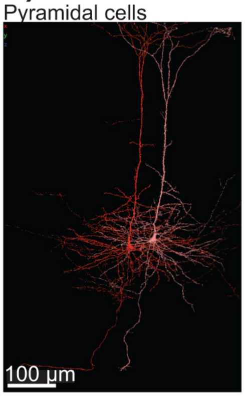

Martinotti cell

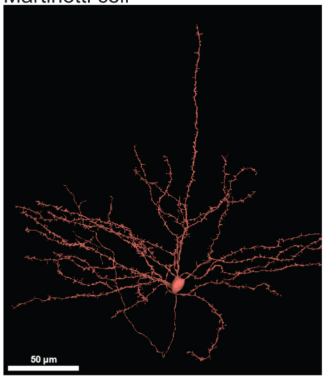

Oligodendrocyte precursor cell

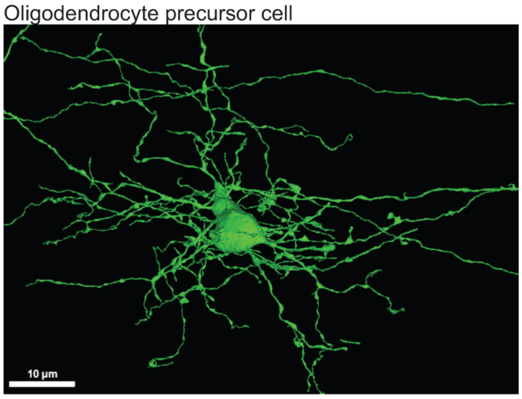

Chandelier cell

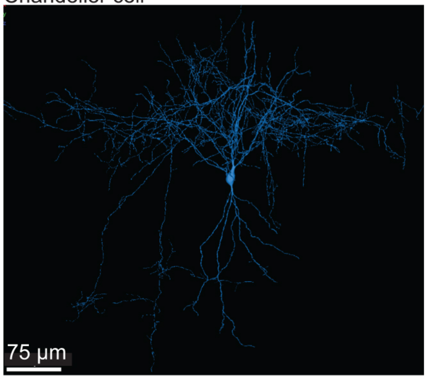

White matter axons

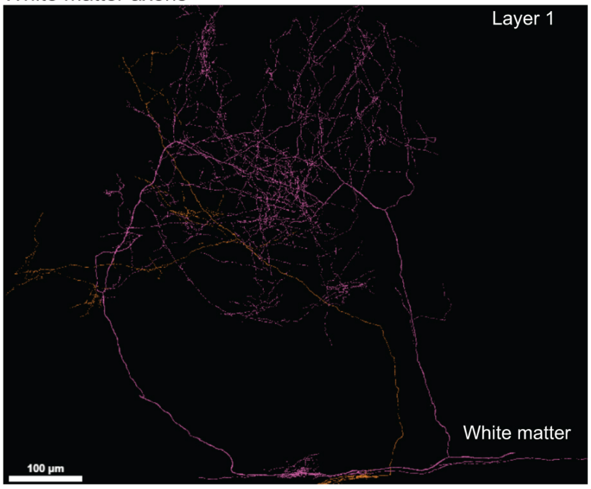

Supplement: Supplementary Figure 3 [file supplementary_figure_3.pdf]
