# Supplementary material for: Ultrastructure of precapillary sphincters and the neurovascular unit
Source: Vasc Biol. 2023 Dec 1;5(1):e230011. doi: 10.1530/VB-23-0011 (PMC10762554; doi:10.1530/VB-23-0011)

Supplementary Figure 4

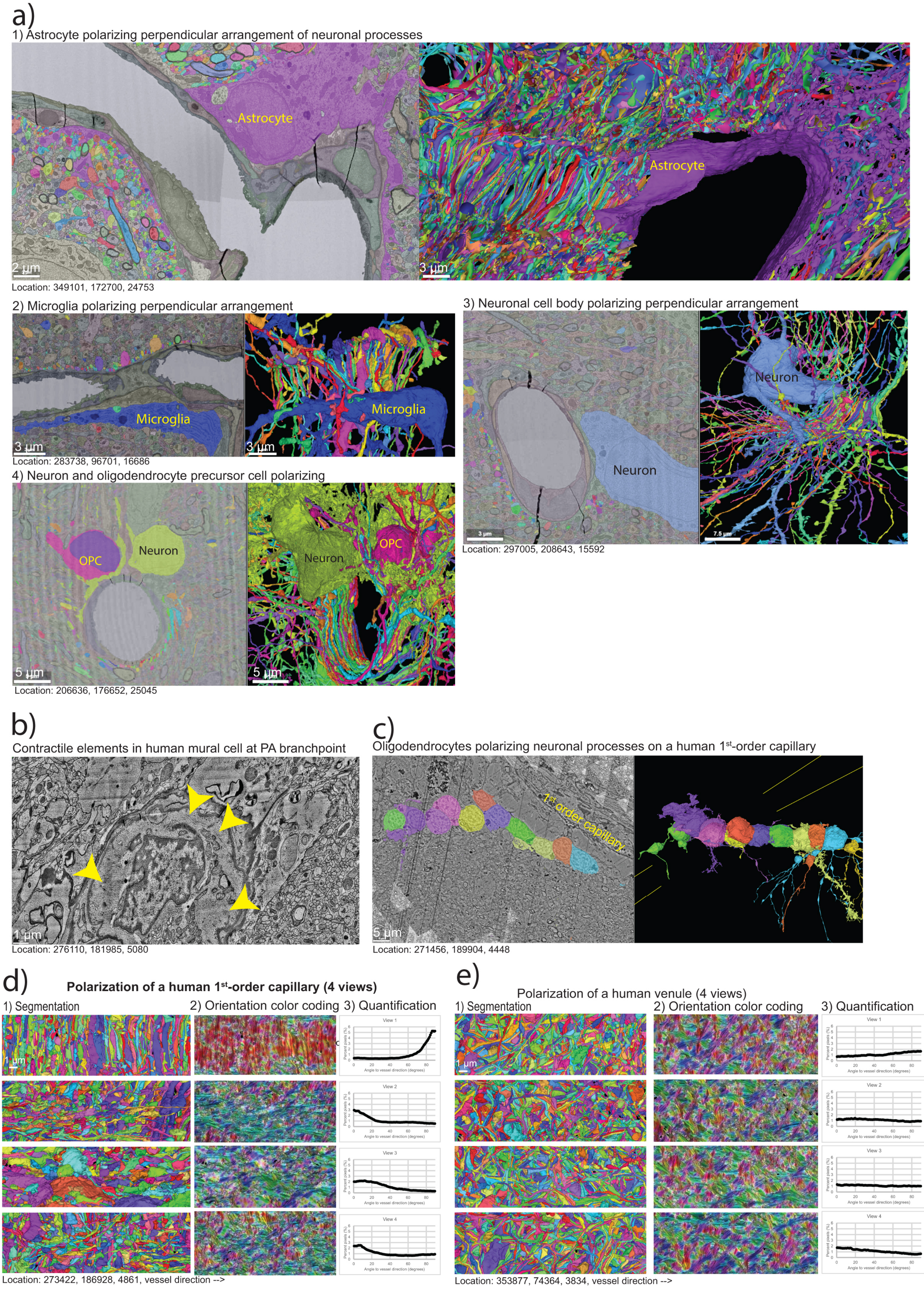

Supplement: Supplementary Figure 4 [file supplementary_figure_4.pdf]
