# Supplementary material for: Ultrastructure of precapillary sphincters and the neurovascular unit
Source: Vasc Biol. 2023 Dec 1;5(1):e230011. doi: 10.1530/VB-23-0011 (PMC10762554; doi:10.1530/VB-23-0011)

Supplementary Table 1 – quantification of precapillary sphincters:

Arteriole#	Branch#	Coordinates	Sphincter Ø	1. Order Ø	Ratio	Classification
**1**	1	74595, 108426, 19742	3.58	7.14	0.50	Sphincter
	2	75268, 117870, 19100	4.98	6.52	0.76	Sphincter
	3	72732, 153978, 19551	4.84	6.25	0.77	Sphincter
**2**	1	170006, 165066, 19289	7.72	7.98	0.97	Not Sphincter
	2	173800, 173543, 19554	4.67	5.94	0.79	Sphincter
	3	173787, 212162, 18394	NA	NA	NA	Arteriole
	4	173755, 209460, 17937	NA	NA	NA	Arteriole
	5	172137, 206164, 17850	2.97	3.28	0.91	Not Sphincter
	6	168645, 199074, 17966	5.80	6.80	0.85	Not Sphincter
**3**	1	113960, 92549, 23418	2.39	4.37	0.55	Sphincter
	2	122150, 181802, 22428	NA	NA	NA	Arteriole
	3	121444, 184487, 22724	7.91	7.39	1.07	Not Sphincter
	4	121747, 195362, 23039	3.18	3.92	0.81	Not Sphincter
**4**	1	210284, 109979, 25409	NA	NA	NA	Bifurcation
	2	204773, 163891, 24599	6.04	6.36	0.95	Not Sphincter
	3	202486, 172266, 24849	4.93	5.72	0.86	Not Sphincter
	4	204976, 179241, 24959	8.86	8.64	1.02	Not Sphincter
	5	212398, 190774, 24360	4.70	6.10	0.77	Sphincter
	6	210644, 204189, 24922	6.16	6.06	1.02	Not Sphincter
	7	206126, 207593, 24682	7.36	7.04	1.05	Not Sphincter
	8	196045, 207297, 24256	2.15	2.54	0.85	Not Sphincter
**5**	1	357846, 94680, 25093	4.95	7.24	0.68	Sphincter
	2	357048, 106431, 25451	4.77	5.65	0.84	Not Sphincter
	3	359213, 106537, 24991	6.81	8.93	0.76	Sphincter
	4	351485, 169041, 25054	3.76	4.11	0.92	Not Sphincter
	5	348820, 173983, 24917	5.22	5.65	0.92	Not Sphincter
	6	339946, 194450, 25441	4.71	4.84	0.97	Not Sphincter
	7	340570, 204125, 25050	NA	NA	NA	Bifurcation
	8	335837, 212486, 25656	3.26	3.67	0.89	Not Sphincter
	9	333938, 215257, 25680	3.59	4.04	0.89	Not Sphincter
	10	331871, 227197, 25736	6.72	6.34	1.06	Not Sphincter
**7**	1	225627, 153955, 19298	5.80	7.98	0.73	Sphincter
	2	231199, 199952, 20491	6.14	6.88	0.89	Not Sphincter
	3	236667, 207676, 19947	8.24	8.32	0.99	Not Sphincter
	4	234677, 213898, 20550	NA	NA	NA	Arteriole
	5	233765, 214506, 20427	11.18	8.48	1.32	Not Sphincter
**8**	1	286906, 97518, 16687	3.36	3.12	1.08	Not Sphincter
	2	283959, 126535, 17497	NA	NA	NA	Arteriole
	3	286643, 131713, 17327	NA	NA	NA	Arteriole
	4	284275, 141142, 17436	3.55	3.20	1.11	Not Sphincter
**9**	1	236463, 129244, 16871	6.62	5.62	1.18	Not Sphincter
	2	241777, 132659, 17301	NA	NA	NA	Arteriole
	3	246791, 154761, 18087	3.47	4.17	0.83	Not Sphincter
	4	254674, 158598, 18859	4.45	4.45	1.00	Not Sphincter
	5	256219, 158816, 19143	4.61	4.91	0.94	Not Sphincter
**10**	1	203857, 103574, 15476	3.30	4.22	0.78	Sphincter
	2	204554, 134469, 15410	5.81	4.34	1.34	Not Sphincter
	3	200877, 137255, 15208	NA	NA	NA	Bifurcation
**11**	1	316214, 87729, 16498	3.12	3.61	0.87	Not Sphincter
	2	316857, 100964, 17635	4.65	6.35	0.73	Sphincter
	3	314010, 112441, 17739	4.23	5.45	0.78	Sphincter
	4	317051, 149938, 18634	3.90	3.92	0.99	Not Sphincter
	5	311308, 158316, 18465	NA	NA	NA	Arteriole
**12**	1	153011, 97361, 23487	3.89	4.19	0.93	Not Sphincter
	2	154632, 96935, 23378	3.48	4.64	0.75	Sphincter
	3	156408, 155386, 23591	NA	NA	NA	Arteriole
	4	157740, 160353, 23861	4.06	4.43	0.92	Not Sphincter

	5	159002, 167789, 23673	4.79	5.05	0.95	Not Sphincter
	6	160038, 176438, 23683	NA	NA	NA	Bifurcation
13	1	311988, 189957, 14564	NA	NA	NA	Arteriole
	2	307513, 190698, 13989	NA	NA	NA	Bifurcating
	3	297372, 200369, 15150	4.42	6.77	0.65	Sphincter
	4	293972, 207619, 15405	3.75	5.75	0.65	Sphincter
	5	285692, 214274, 14962	5.42	6.73	0.80	Not Sphincter
14	1	324970, 136938, 11893	NA	NA	NA	Arteriole
	2	326803, 147409, 12565	2.86	4.99	0.57	Sphincter
	3	336099, 168261, 12430	5.11	7.02	0.73	Sphincter
15	1	232832, 101719, 8418	3.57	4.66	0.77	Sphincter
	2	229982, 132612, 8198	4.67	5.02	0.93	Not Sphincter
	3	230329, 139604, 8176	4.43	4.55	0.97	Not Sphincter
	4	229868, 147087, 8382	3.29	2.95	1.11	Not Sphincter
16	1	117487, 101356, 8596	2.65	3.25	0.82	Not Sphincter
	2	121799, 110848, 8747	3.14	3.21	0.98	Not Sphincter
	3	120183, 115464, 8974	NA	NA	NA	Arteriole
17	1	91516, 146482, 10923	NA	NA	NA	Arteriole
	2	99013, 187604, 10661	6.44	6.55	0.98	Not Sphincter
	3	101478, 198669, 11548	4.15	5.60	0.74	Sphincter
	4	108500, 208824, 11448	5.94	5.16	1.15	Not Sphincter
	5	114477, 217696, 11970	4.68	4.89	0.96	Not Sphincter
	6	114477, 217696, 11970	4.66	5.03	0.92	Not Sphincter
18	1	229089, 91841, 13522	NA	NA	NA	Arteriole
	2	230532, 133200, 13774	5.04	4.84	1.04	Not Sphincter
	2	235692, 93125, 12861	5.35	4.74	1.13	Not Sphincter
	3	241644, 91523, 12894	1.53	3.58	0.43	Sphincter
	3	229749, 144984, 13996	4.80	5.53	0.87	Not Sphincter
	4	232541, 159579, 14209	NA	NA	NA	Bifurcation
	5	229886, 167626, 14318	6.33	6.01	1.05	Not Sphincter
	6	232026, 181203, 14557	4.74	5.04	0.94	Not Sphincter
19	1	211221, 104394, 14668	4.74	4.37	1.09	Not Sphincter
	2	212002, 135493, 14535	5.06	5.40	0.94	Not Sphincter
20	1	170378, 112436, 8536	2.47	3.46	0.71	Sphincter
	2	169601, 136370, 8999	4.88	4.89	1.00	Not Sphincter
	3	174295, 154198, 9048	NA	NA	NA	Arteriole
	4	173463, 159538, 9883	5.71	5.19	1.10	Not Sphincter
	5	167585, 164896, 10445	4.71	4.73	1.00	Not Sphincter

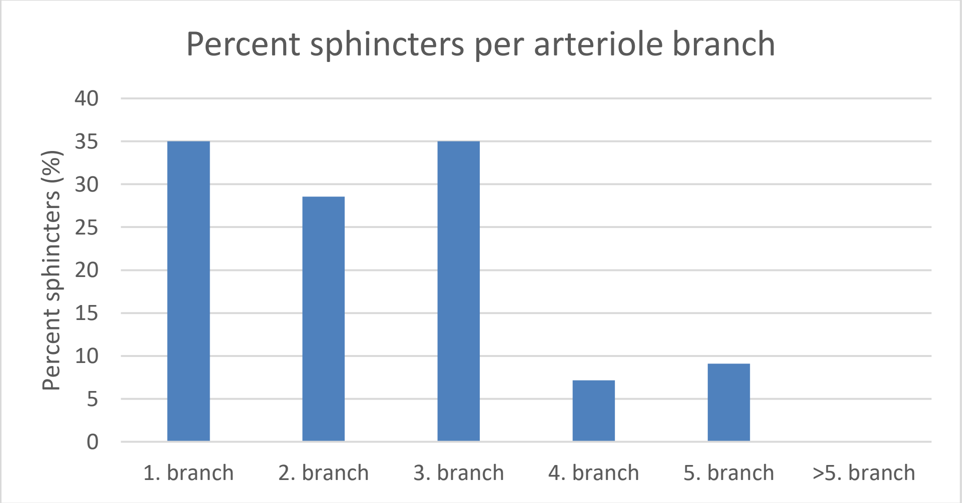

Supplement: Supplementary Table 1 – quantification of precapillary sphincters: [file supplementary_table_1.pdf]
